# Pregnancy following robot-assisted laparoscopic bilateral endometriotic cystectomy rare case report of endometriosis stage IV

**DOI:** 10.1016/j.ijscr.2022.107508

**Published:** 2022-08-13

**Authors:** Willbroad Kyejo, Allyzain Ismail, Brenda Moshi, Gregory Ntiyakunze, Nancy Matillya, Munawar Kaguta

**Affiliations:** a Department of Family Medicine, Aga Khan University, P.O. Box 38129, Dar Es Salaam, Tanzania; b Department of Surgery, Aga Khan University, P.O. Box 38129, Dar Es Salaam, Tanzania; c Department of Obstetrics and Gynecology, Aga Khan Hospital, P.O. Box 2289, Dar Es Salaam, Tanzania

**Keywords:** Endometriosis stage IV, Robotic laparoscopic endometriotic cystectomy, Case report

## Abstract

**Introduction and importance:**

Endometriosis is a common cause of infertility in women. In this case report we explain successful conception in deep-infiltrating ovarian endometriosis following robot-assisted surgery and androgenic agonist treatment.

**Case presentation:**

A 38-year-old current Para 2, Living 2 presented 8 years ago with chronic lower abdominal pain, dysmenorrhea, and delayed conception. Advance endometriosis was highly suspected from the history and examination. Robot-assisted laparoscopic partial cystectomy was performed for the deep-infiltrating ovarian endometriosis. She was then discharged with postoperative androgenic agonists and with timed intercourse, she got pregnant within 9 months.

**Clinical discussion:**

Advanced endometriosis (Stage III or IV disease) is associated with distorted pelvic anatomy and adhesions. These changes can impair oocyte release or pick-up, alter sperm motility, cause disordered myometrial contractions, and impair fertilization and embryo transport. Successful rate of conception in advanced endometriosis is <20 %, with minimal chances of successful conception, however, our patient conceived.

**Conclusion:**

Endometriosis should be considered in evaluation of reproductive woman with chronic lower abdominal pain, history of infertility and dysmenorrhea. Prompt management is crucial, and in a setting of advanced technology, robotic laparoscopic surgery is the best to diagnosis and treat advanced endometriosis.

## Highlights

• Successful pregnancy after laparoscopic endometriotic cystectomy
• Lady with infertility and chronic lower abdomen pain
• Multidisciplinary approach

## 1 Introduction and importance

Endometriosis is the presence of endometrial glands and stroma at extrauterine sites throughout the pelvis and beyond [1]. Endometriotic lesions can be superficial or deep. The lesions that invade into the rectovaginal space and/or bowel are forms of deep infiltrating endometriosis [1]. The invasive nature of these implants causes significant pelvic pain, dyspareunia, bowel dysfunction, and results into treatment challenge [1].

The prevalence of endometriosis has been reported in up to 50 % of infertile women [2]. Distorted pelvic anatomy secondary to pelvic adhesions and increased inflammation adversely impacting the peritoneal environment contribute to endometriosis-associated infertility [2], [3]. Deep-infiltrating endometriosis (DIE) is a complex form of endometriosis which may involve ovaries, rectovaginal septum, uterovesical fold and bladder. It is believed that DIE has more aggressive behavior and more detrimental effects on fertility in comparison to superficial peritoneal endometriosis [4]. Both the American Society for Reproductive Medicine and the European Society of Human Reproduction and Embryology (ESHRE) recommend operative laparoscopy to increase spontaneous pregnancy rate for women with stage III/IV endometriosis [2], [3], [5]. However, there is no consensus about the therapeutic strategies of DIE to improve fecundity [5].

We report a case of a woman with history of chronic lower abdominal pain, dysmenorrhea and delayed conception that was diagnosed to have endometriosis stage IV and underwent robotic laparoscopic endometriotic cystectomy successfully for relief of her symptoms followed with androgenic agonist. In this case report from Aga Khan Hospital, Dar-es-Salaam, Tanzania, we describe our approach to diagnosing and treating endometriosis stage IV with challenges faced due to lack of resources and expertise. This paper has been reported in line with the SCARE 2020 criteria [6]. This article has been registered with the Research Registry with identification number researchregistry7781 and can be found through the following hyperlink Browse the Registry - Research Registry.

## 2 Case presentation

38 years old lady current Para 2, living 2, presented at our centre 8 years ago with history of recurrent lower abdominal pain since menarche (Attained at 11 years of age). It was of intermittent dull aching in nature, non-radiating, more severe on bending and squatting. It was severe to the extent that it was interfering with her daily working activities. It was relieved with resting and massaging the abdomen. The pain was severe during menstruation to the extent that she would require intravenous analgesia to relieve the pain, her illness was associated with irregular menstruation and heavy menses bleeding require her to change 4–5 pads per day which were fully soaked. At times it was accompanied with awareness of her heartbeat and easy fatigability. She also reported to have experienced very painful sexual intercourse, which was one of the aggravating factors of her chronic lower abdominal pain.

During her illness, she has been trying to conceive for the last 4 years without any success, her husband has done multiple semen analysis with normal results.

Her illness was accompanied with loss of interest and having low mood at times to extent that she has been seeing psychologist for minor depression episode. She reports no history of painful urination, no change in bowel habit, no history fever and no blood in stool or difficult defecating.

She has a history of open appendectomy in 2005, however no history of chronic illness or significant family history of similar disease/symptoms, no drug allergies and did not smoke or drink alcohol.

On examination she was alert, not pale, no lower limb edema and had stable vital signs. Per abdomen revealed mild distension, gridiron's incision scar, no obvious mass palpable, mild tenderness on Suprapubic region with no shifting dullness and rest of systemic examinations were normal.

Initial work up was done in outpatient gynecology clinic and results were CA-125, CA19-9 and CEA of 2.77 ng/ml, 20.81 U/ml, 37.65 U/ml and 2.05 ng/ml, respectively. Initial ultrasound showed fluid filled bowel loops noted with increased peristaltic movement and minimal interloop collection, bilateral ovarian cyst, right side measuring 6 × 4 cm and left side measuring 5 × 3 cm each adhered to the posterior surface of uterus together with rectum. Uterus had a small intramural myoma in the lower posterior wall. Bilateral fallopian tubes were inflamed, urinary bladder appeared normal, features suggestive of advanced Endometriosis (Stage IV) with partial intestinal obstruction.

Her partial intestinal obstruction was treated conservative by medical therapy for 3 days in the ward then she was counseled for diagnostic laparoscopic and biopsy at our centre, however patient opted to travel to a hospital in India for second opinion to undergo robotic surgery since she had read in many websites it carries less morbidity and less hospital stay.

Initial work ups were done as shown below.

**Table:** 

TEST	Results	Range
Anti-Mullerin Hormone (AMH)	2.12 ng/ml	0.777–5.24 ng/ml
CA (Cancer Antigen) 125-serum	**41 U/ml**	<21 U/ml
Glycated hemoglobin	5.0 %	<5.7 %
Prolactin	**56.5 ng/ml**	1.9–25 ng/ml
Testosterone	15.53 ng/dl	65–119 ng/dl
Total T3 (Tri iodothyronine)	74 ng/dl	80–120 ng/dl
Total T4: Thyroxines	7.8 ng/dl	5.1–14.1 ng/dl
Hemoglobin	11.2 g/dl	11.0–16.0 g/dl

**Fig. 1:**
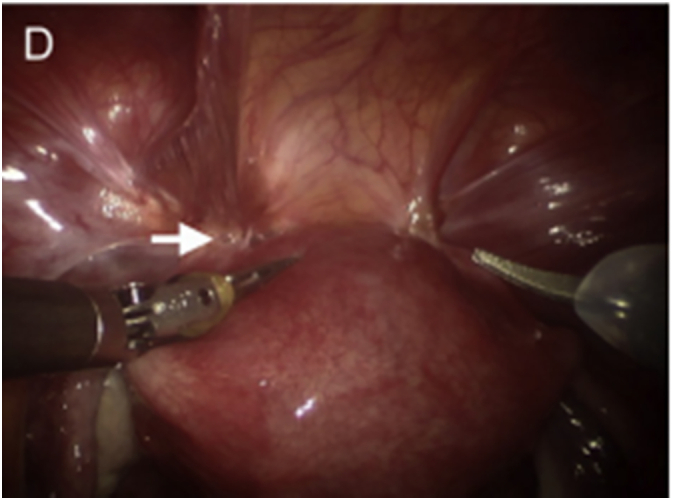
Laparoscopy revealed extensive deep infiltrating endometriosis with obliterated uterovesical fold (white arrow).

Based on laboratory results above together with sonographic features, we had diagnosis of Advanced Endometriosis, with differential diagnosis of severe adenomyosis. Then patient consented for diagnostic robotic-laparoscopy bilateral cystectomy for tissue biopsy and possible adhesiolysis. Under aseptic presenting, Robotic arms introduced through abdominal ports and findings were two bilateral endometriotic cysts present, right side measures 8 × 8 × 8 cm, Left side measures 5 × 3 × 3 cm adhered with each other to the posterior surface of uterus up to fundus (Fig. 1).

Small adenomyoma fibroid in lower segment posterior myometrium, rectum adherent to posterior wall of uterus and bilateral fallopian tubes were inflamed.

**Fig. 2:**
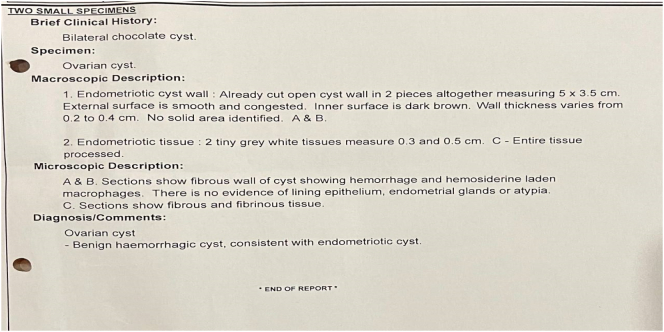
Histology report from ovarian cysts.

The following was done both endometriotic cysts were punctured, chocolate fluid aspirated, cyst wall excised and sent for histopathology. Extensive adhesiolysis done and small endometriotic spots present on post surface of uterus were coagulated. The rectum was dissected from the posterior uterine wall. Hemostasis was achieved. Chromopertubation was done, left side free spill of dye present, right side had delayed spill of dye. Hemostasis reconfirmed, the estimate blood loss was <50 mls. Port closure done. Her histopathology report confirmed endometriosis (Fig. 2). The procedure was done by highly Gynecologist experinced in laparoscopic and robotic surgery.

Post-operative patient was kept on IV paracetamol 1 g 8 hourly for 3 days, IM pethidine 100 mg as per required and IV ceftriaxone 1 g 12 hourly for 3 days.

She was discharged day 4 post-operative with the following medication Tabs olanzapine 2.5 mg nocte for 14 days (Depression), Tabs Ovares 25 mg 8 hourly after food for 2 months (androgenic agonist to enhance ovulation), Tabs Folic acid 5 mg daily 30 days and Tabs multivitamin 1 tab daily for 30 days. And patient was encouraged to have timed sexual intercourse monthly.

In view of surgical findings, endometriosis stage IV was suspected, and conception was unlikely however after consecutive follow up every monthly, patient was able to conceive after 9 months of using above medication, she reported during pregnancy her pelvic pain was reduced significantly, and she was able to deliver 3.2 kg male baby in 2018 and again in early 2020 she was able to conceive naturally without assistance of medication and deliver her second child 2.8 kg by cesarean section.

## 3 Discussion

Advanced endometriosis (Stage III or IV disease) is associated with distorted pelvic anatomy and adhesions. These changes can impair oocyte release or pick-up, alter sperm motility, cause disordered myometrial contractions, impair fertilization and embryo transport [7].

It also negatively impacts the ovarian follicles maturations. Compared with women who have mild endometriosis or tubal factor infertility, studies on women with advanced endometriosis have reported abnormal folliculogenesis and reduced fertilization potential of oocytes [8].

A multicentric study found that 21 % of patients with DIE were infertile and the mean duration of infertility was 3 years [9]. Laparoscopic surgery for the treatment of endometriosis has been proven effective in reducing pain and increasing live birth or ongoing pregnancy rate (OR 1.94, 95 % CI 1.20 to 3.16) and clinical pregnancy rate (OR 1.89, 95 % CI 1.25 to 2.86) compared with diagnostic laparoscopy [10]. Robotic laparoscope is recommended for infertile women with stage III/IV endometriosis without other identifiable infertility factors which is similar in our case.

A balance between the benefits and the risks of aggressive surgery should be weighed. The revolution of robotic technology offers surgeons improved ergonomics, three-dimensional visualization, greater precision, fine instrumentation, and a shorter learning curve and overcomes the limitations of conventional laparoscopy. Also it shows superiority on Gynecological procedures [11]. This became a challenge in most of Sub-Saharan African countries including Tanzania due to the lack of advanced equipment and we opt to do open surgery (laparotomy) which carries great risk of adhesions and iatrogenic injury. In this case our client was able to travel to Apollo Health city Campus hospital (India) in which there was availability of these advanced equipment.

Another multicentric study retrospectively investigated the perioperative outcomes in patients with Deep Infiltrated Endometriosis undergoing robot-assisted laparoscopy [9]. The study included 164 patients, and 74 patients (45.1 %) needed multiple surgical procedures for at least two locations of DIE. In the 13 cases of ovarian endometriosis and posterior wall bladder endometriosis, the mean operative time was 207.2 ± 85.5 min, and estimate blood loss was 57.6 ± 251.8 ml. Which this is like our case the blood loss was minimal however duration taken for the surgery was not documented.

Further study have shown overall pregnancy rate is 41.9 %, 66.7 % and 94.4 % of the patients conceived within postoperative 3 months and 6 months, and 9 months respectively. The spontaneous pregnancy rate was not associated with the severity of endometriosis or laparoscopic findings or the type of surgery. However those with Endometriosis stage IV had relatively low 20 % chances of conception compared to endometriosis stage I, II, and III (35.7 %, 44.4 %, and 53.3 %, respectively) [12]. In our patient the chance of conception was low, but she was able to conceive twice with good neonatal outcome.

## 4 Conclusion

Endometriosis should be considered in evaluation of reproductive woman with chronic abdominal pain, history of infertility and dysmenorrhea. Prompt management is crucial, and depending on the available resources, multidisciplinary approach is of paramount importance.

## Abbreviations

AFP: alpha feto protein
CT: computed tomography
IV: intravenous
IM: intramuscular
USS: ultra sound scan
APGAR score: Appearance, Pulse, Grimace, Activity, and Respiration score

## Patient perspectives

I had visited multiple centers and given many herbals and antibiotics which didn't help me in terms of conceiving. In some centre in my country was counseled for hysterectomy because chronic lower abdominal pain but once I underwent robotic laparoscopic cystectomy, I had a relief in terms of the pain but still was uncertain in becoming pregnant.

Conceiving and delivering two times to me was a surprise given little amount of hope that I had.

## Provenance and peer review

Not commissioned, externally peer-reviewed.

## Funding

No funding was provided for research.

## Ethical approval

Case study is exempt from ethical approval in my institution.

## Consent

Written informed consent was obtained from the patient for publication of this case report and accompanying images. A copy of the written consent is available for review by the Editor-in-Chief of this journal on request.

## Author contribution

W.K: Study conception, production of initial manuscript, collection of data

A.I: Production of initial manuscript, revision of the manuscript, proofreading

B.M: Revision of the manuscript, proofreading

G.N: Revision of the manuscript, proofreading

N.M: Revision of the manuscript, proofreading

M.K: Study conception, revision of the manuscript, proofreading

## Registration of research studies

1. Name of the registry: RESEARCH REGISTRY
2. Unique identifying number or registration ID: researchregistry7781
3. Hyperlink to your specific registration (must be publicly accessible and will be checked): Hyperlink

## Guarantor

Dr. Munawa Kaguta.

## Declaration of competing interest

No conflicts of interest.
